# Determinants of Early Marriage from Married Girls' Perspectives in Iranian Setting: A Qualitative Study

**DOI:** 10.1155/2016/8615929

**Published:** 2016-03-30

**Authors:** Simin Montazeri, Maryam Gharacheh, Nooredin Mohammadi, Javad Alaghband Rad, Hassan Eftekhar Ardabili

**Affiliations:** ^1^Department of Reproductive Health, School of Nursing and Midwifery, Tehran University of Medical Sciences, Tehran, Iran; ^2^Department of Critical Care Nursing, Centre for Nursing Care Research, School of Nursing and Midwifery, Iran University of Medical Sciences, Tehran, Iran; ^3^Department of Child and Adolescent Psychiatry, School of Medicine, Tehran University of Medical Sciences, Tehran, Iran; ^4^Department of Health Education, School of Public Health, Tehran University of Medical Sciences, Tehran, Iran

## Abstract

Early marriage is a worldwide problem associated with a range of health and social consequences for teenage girls. Designing effective health interventions for managing early marriage needs to apply the community-based approaches. However, it has received less attention from policymakers and health researchers in Iran. Therefore, the current study aimed to explore determinants of early marriage from married girls' perspectives. The study was conducted from May 2013 to January 2015 in Ahvaz, Iran. A purposeful sampling method was used to select fifteen eligible participants. Data were collected through face-to-face, semistructured interviews and were analyzed using the conventional content analysis approach. Three categories emerged from the qualitative data including “family structure,” “Low autonomy in decision-making,” and “response to needs.” According to the results, although the participants were not ready to get married and intended to postpone their marriage, multiple factors such as individual and contextual factors propelled them to early marriage. Given that early marriage is a multifactorial problem, health care providers should consider a multidimensional approach to support and empower these vulnerable girls.

## 1. Introduction

Adolescence is a critical age for girls throughout the world. What takes place during a girl's teenage years shapes future life circumstances [1]. For many girls in the developing world, the mere commencement of puberty marks a time of increased vulnerability to early marriage [2] and entry into sexual life [3]. Thus, marriage is central to any discussion in the area of the sexual and reproductive health of adolescents in the Arab countries and Iran “because of the universal valuation of marriage and the taboos and religious sanctions against premarital and extramarital sexual relations” [4].

Today, there are estimated 580 million teenage girls in the world of whom 88 percent live in the developing countries [3]. While the age of marriage is rising for both sexes, early marriage has still remained a problem in these societies. Early marriage as a global issue and a widespread harmful practice affects enormous numbers of girls [5]. According to United Nations Population Fund, nearly one in three girls continues to marry as a teenager in many parts of the developing world [2]. The extent of early marriage varies between countries and regions. The highest rates are reported in South Asia and Sub-Saharan Africa, where 44 percent and 39 percent of girls, respectively, were married before the age of 18. According to data from 33 countries, trends in marriage indicate limited change since the International Conference on Population and Development (ICPD) [3]. Iran is no exception in this regard. Iranian studies indicate that more than 7.7 percent of girls in Tehran and 40 percent in Sistan and Baluchestan were married before the age of 18. The rate of teen early marriage in rural and urban areas has been reported as 19.6 percent and 13.7 percent, respectively [6]. According to the latest census in Iran, the highest number of registered marriage pertained to women who were 15–19 years of age (129780 events) [7].

There are various factors contributing to the existence of early marriage including male dominancy, parent's ignorance, and social forces [8]. Early marriage is most likely to occur among girls who are poor, have low education level, and live in rural areas [2]. It denies girls opportunities to educate and to build life skills, separates them from family and friends, compromises their ability to assume health promotion practices and seek timely care, and enhances their vulnerability to considerable health and social problems [5, 9–11].

Recent evidence argues that early marriage can be associated with adverse consequences not only for teenage girls but also for children they bear [11]. High rates of unintended pregnancy, abortion, preterm labor, delivery of low birth weight babies, and fetal and maternal mortality are observed among teenage girls and are strongly correlated with early marriage [3, 10]. Moreover, the girls who are married as teenagers are also affected psychologically and they are more likely to experience depression, anxiety, and other mood disorders [8]. They are especially at risk for physical and sexual violence within marriage [5]. WHO reports that 29 percent of all ever partnered teenage girls experience intimate partner violence. The harmful impacts of domestic violence on the health and wellbeing of women and their children are well documented [3]. Thus, teenage girls are one of the most vulnerable and neglected individuals in the world and investing in them is important for ensuring health, creating prosperity, and fulfilling human rights. Addressing the needs of adolescents, particularly teenage girls, is necessary not only for their individual profit, but also to attain global goals such as reducing maternal mortality and HIV infection [5].

Early marriage and parenthood are encouraged by Iranian culture. In Iran, puberty and menarche are considered as time of transition to adulthood. Girls reaching this biological threshold means becoming eligible for marriage, regardless of age [12]. According to Iranian civil law, the legal age of marriage is set at thirteen for girls and fifteen for boys [6].

Socioeconomic and cultural factors are the main determinants of age at marriage in Iran. Poverty that compels parents to marry off their daughters, parents' tendency for the bride price (mahrieh), social and political ties, women's low socioeconomic status, and religious beliefs prohibiting extramarital sex have been identified as reasons for girls' early marriage in Iran [12]. A study by Matlabi et al. also revealed that the most important factors of early marriage in rural areas were traditional issues and cultural poverty, low awareness of girls and their parents about the risk of child marriage, negative attitude towards continuation of education, freedom from undesirable and rigid rules of parents, and low authority of girls and lack of power to make decision [6].

In Iran, early arranged marriage was very common in the past, and mate selection was mostly determined by parents and confirmed by kinship system. In the recent decades, early arranged marriage has still remained common in certain parts of the country regardless of the wide acceptance of modern familial values, norms, and attitudes toward marriage. In Iran as a multiethnic society, marriage patterns are different among ethnic groups [13]. Torabi and Baschieri illustrated that socioeconomic characteristics of ethnic groups differently influence the timing and probability of Iranian women's marriage [14].

Iran has witnessed fundamental economic, social, political, and cultural changes in the past three decades [13]. These structural changes have led ethnic groups to experience different levels of modernization and development, while maintaining their distinct cultural ethnic norms and values [14]. This condition could fuel norms and values reinforcing early marriage [13]. Although modernity has affected many aspects of human life, perspectives towards early marriage have not kept pace with this change. Economic hardship and an increase in poverty, mentioned as key determinants in the continuation of early marriage, may have reinforced the attitudes towards early marriage, particularly in some parts where family ties have been affected by this change [15].

In transition to modernity, regardless of recent general trends identified as being rising age at marriage, early marriage has still remained a problem in most societies [15] and has not received sufficient attention by researchers [16, 17]. Therefore, there is immense need to undertake research on deeper understanding of the determinants of early marriage from girls' own perspectives. It will contribute to designing and developing culturally responsive interventions and improving the health programs for these girls. Given the importance of early marriage and its impacts on health of adolescents and the role of culture as the main determinants of age at marriage in Iran, this qualitative study was carried out to explore determinants of early marriage from married girls' perspectives in Iranian setting.

## 2. Methods

A qualitative inquiry with content analysis approach was chosen as a research methodology. It is the suitable method to explore cultural context-bound subjects about which there is little knowledge [18]. Qualitative content analysis is a subjective interpretation of the content of text through the systematic process of coding and identifying themes or categories [19] to reach a broad and condensed description of the study phenomenon [18]. Qualitative content analysis consists of three approaches including conventional, directed, and summative [19]. In the current study, the conventional approach was employed.

It must be noted that this paper is part of a larger qualitative study exploring motivations, perceptions, beliefs, values, and attitudes of married girls toward early marriage and, in this paper, the determinants of early marriage are presented as a part of those study findings.

### 2.1. Participants and Setting

This study was conducted from May 2013 to January 2015 in Ahvaz, Iran. Fifteen participants who attended the health care centers were selected through the purposeful sampling method. For this purpose, the principle author approached the potential participants. Each participant who had inclusion criteria is provided with information about the research and encouraged to participate in the study. Inclusion criteria were married girls, aged 3–19 years; speaking Persian language; living in Ahvaz geographical border; and willing to participate in this study. The demographic characteristics of participants are presented in Table 1.

### 2.2. Data Collection

The main technique for data collection was interview. Interviews were conducted by a single researcher who is Ph.D. candidate of sexual and reproductive health with over 25 years of educational and clinical experiences in maternal child health care. The data was driven from 18 semistructured, in depth, and face-to-face interviews with fifteen participants. For obtaining more information, three participants had two interview sessions. Sampling continued until saturation of data was reached. All participants were interviewed at a private room in the health care centers. The participants were asked to talk about the conditions that propelled them to early marriage. The interviews were begun with a general question: “Could you tell me about your marriage?” Probing questions also were used to clarify participants' descriptions, such as “When you said… what did you mean?, Could you explain more about that?” Duration of the interview sessions varied from 45 to 90 minutes. All interviews were audiorecorded in MP3 format and transcribed verbatim in Persian language. Then, each transcript was saved to a separate Rich Text Format file and imported to MAXQDA 10 software.

### 2.3. Data Analysis

The conventional content analysis approach was applied to analyze the data using the method described by Graneheim and Lundman [20], as follows. All interviews were read several times to gain a sense of the whole. The transcripts were divided into condensed meaning units that were abstracted and coded. Then, codes were compared according to similarities and differences and sorted into categories and subcategories constituting the manifest content. The tentative categories were revised by the research team members. Finally, categories were formulated as the latent content of the text. In addition, all transcripts were translated from Persian language to English language by the authors who were fluent in both English and Persian languages.

### 2.4. Trustworthiness

In the current study, Lincoln and Guba's guideline [21] was followed to ensure trustworthiness. Credibility was established through choosing a capable research approach and suitable research team, applying purposeful sampling to select appropriate participants, prolonged engagement with participants and data, member checking and peer reviewing to verify the universality of the findings, and employing triangulation with multiple data collection such as field notes and participant diaries. To assure dependability and confirmability, the external audit trail was done. In addition, a precise final report was provided on the research process to enhance transferability.

### 2.5. Ethical Consideration

Ethical approval was obtained from the Ethics Committee of the Research Deputy in Tehran University of Medical Sciences. The participants were informed about the purpose of the study, and written informed consent was signed by each participant and her husband for permission to participate in the study. Also, participants' names were changed to pseudonyms to maintain the anonymity.

## 3. Results

Three categories were extracted from the deep descriptions of the participants including “family structure,” “low autonomy in decision-making,” and “response to needs.” These categories consisted of ten subcategories (Table 2).

### 3.1. Family Structure

Family structure as the first category refers to socioeconomic difficulties, cultural family values, and religious beliefs that propelled the study participants to early marriage.

#### 3.1.1. Socioeconomic Difficulties

Most participants had grown up in dysfunctional family with socioeconomic difficulties. They had experienced difficult and excruciating childhood. This was a fundamental reason that persuaded them to get married.

*“I had a difficult childhood because my father had died and my family was poor. I could not get everything that I would like to have. I had a big and extended family. My mother was not able to take care of us at all. We [my sisters and I] could not tolerate this difficult situation. My mother wanted to get rid of her responsibilities toward her children, so I thought if I get married, everything will be better.”* (p: 2)

Another participant added,* “Marriage was the best way that I could get rid of my family problems.”*

#### 3.1.2. Cultural Family Values

According to the participants, one of the main factors influencing their marriage-related decision-making was traditional and cultural family values. One of the participants pointed out,

*“People think when a girl grows up physically, she is ready for marriage”* (p: 4).* “My family believed that it's better for girls to get married early. My sisters all got married before the[sic] turned 16 too.”* (p: 13)

Some participants believed that marriage makes people become more mature and responsible. The following excerpts were taken from two participants.

*I wanted to marry because l thought if I get married, I will become more responsible. I think I know more than my friends who are still single. I have a sense of primacy. I feel I am mature than them.* (p: 6)

*Marriage was very important for my family. My mother said to me I am not a little girl anymore after marriage. I will become more mature mentally than I used to be. I can make more plans for the future.* (p: 10)

From the participants' perspectives, being encouraged to marry by family influenced their marriage-related decision-making process. One of the participants pointed out,* “I was looking for an excuse not to get married, but my parents convinced me by describing all the benefits of marriage. I only agreed to marry in order to please them”* (p: 8). Another participant added,

*My family believed every girl has to get married eventually. They justified it in this way; I didn't want to disobey them. That was why I agreed to marry.* (p: 11)

Spouse's personal situation was another factor that propelled the participants to marry. Regardless of girl' age, if any suitor was morally and economically in good condition, parents would encourage their daughter to marry him: “*My dad told me, the boy is a nice person to marry and I shouldn't reject his proposal”* (p: 15); “*The boy was polite, educated and had a good job. He also had no problem with me to continue my education after getting married. My mom wanted me to marry him*” (p: 6).

The findings showed that consanguinity marriage was very important for the participants and their families. However, if a suitor from relatives had unsuitable conditions, parents would reject him. One of the participants expressed,

*My uncle insisted that I must get married a boy from our relatives, but he was jobless… I had to marry a stranger man who I didn't know him previously but he was in good condition to get married.* (p: 4)

#### 3.1.3. Religious Beliefs

For most participants, marriage-related decision-making was influenced by the adherence to religious beliefs. One of participants explained,* “My mother and grandmother advised me to marry as soon as possible because marriage is one of our prophet's recommendations”* (p: 3). Another participant added,* “My family believed that marriage can protect me from sin [outside of marriage sex]”* (p: 13).

### 3.2. Low Autonomy in Decision-Making

Low autonomy in decision-making due to insufficient life skills including decision-making, problem-solving, negotiation, and critical thinking skills were other reasons for propelling the participants to early marriage.

#### 3.2.1. Inappropriate Decision-Making Skills

Most of the participants believed that they were not able to make appropriate decision concerning their marriage because they could not foresee consequences of their decisions. Therefore, they accepted the marriage-related decisions made by their parents. Following quotations were stated by three participants.

*I told my parents, at my age, I can't make proper decision about marriage, so I leave the decision-making to you because I might make mistake. If you [her parents] confirm [the marriage] I'll agree to it.* (p: 5)

*I had never thought about marriage or the guy who I was going to marry. I couldn't make decision appropriately. It bothered me so much because I was not ready for marriage and had to marry while still being a kid.* (p: 3)

Most participants mentioned that they had not enough information regarding marriage and could not find anyone for consultation to make proper decision. One participant said,* “My parents wanted me to marry. I didn't know what I should do. I didn't have anyone to ask for any advice. I had no choice except to accept my parents' decision”* (p: 11).

Marriage had occurred suddenly and untimely for most participants. For them, marriage was considered as a stressful life event and teenage girls had no sufficient skills for coping with such a stress. They believed that they could not make proper decision related to their marriage under stressful circumstances. In this regard some participants said,* “Everything happened so suddenly that I was unable to think about it logically… at that time, I couldn't justify my parent”* (p: 1).

*I used to be a calm person, but after the marriage proposal, I've got so stressed out that felt like I was losing my balance. It was very surprising for me. Until then, I hadn't thought about marriage at all. I was just a kid, going to school, and had never thought of marriage. Whenever they talk to me regarding marriage, I would get angry and upset. I cried so much and I couldn't do anything.* (p: 9)

#### 3.2.2. Inadequate Problem-Solving Skills

Some participants were pushed toward marriage to escape from school. They had not learned the skills that help them to solve their problems. In* this regard, one participant stated “I was weak in math at school and I didn't like my math teacher, so I didn't like going to school. My dad said that if you wouldn't like to go to school, then you had to marry… To escape from attending classes, I got married*” (p: 8).

#### 3.2.3. Insufficient Negotiation Skills

Most participants expressed that they were not ready psychologically for marriage and its responsibilities. They intended to postpone their marriage until reaching the proper age, as well as achieving their educational and occupational goals. However, they were not able to convince their parents to delay their marriage. In this regard, one of the participants pointed out,

*I had never thought about marriage and the guy who I was going to marry. It was shocking that I was getting married, but my parents didn't care about that I couldn't understand this situation. I really wasn't ready to marry and accept all of its responsibilities while still being a kid.* (p: 7) Another participant said,

*When my family suggested to get married, I cried all day because I was so young. I told my mother I don't like to marry now. I was at first grade of high school at that time; I wanted to finish my education before getting married. But my parents were older than me and I could not convince them to accept my explanations.* (p: 1)

#### 3.2.4. Lack of Critical Thinking Skills

Some participants believed that they were not able to predict and evaluate consequences of early marriage and concerned about the continuation of their marriage. Therefore, they gave marriage-related decision-making over to their parents. One participant stated,

*I'm not sure about marriage consequences. I ask myself whether the marriage is good or not. Can I have a successful life? Can I continue my education? Will the marriage take away my freedom? I told my mother I can't make decision because I can't imagine what will happen in the future… they [my parent] decided for my marriage.* (p: 5)

Another participant said,* “I am not mature enough to evaluate people's mind and behavior yet. I didn't feel well about marriage because I didn't know him [my suitor] well. That is why, I'm afraid that I couldn't get along with him and our marriage ended up in divorce…  I completely gave everything over to my family.* (p: 9)

### 3.3. Response to Needs

According to the participants, marriage was an opportunity to meet some of their social emotional and sexual needs.

#### 3.3.1. Social Needs

The social needs that propelled the participants to early marriage were the needs to receive respect, serenity, and independency. In this regard, two participants stated the following:

*I really needed to have peace in my life, because there were lots of financial and family related problems in my life [living with my parents]. I thought by getting married, I will be relieved of these sufferings.* (p: 2)

*I couldn't get any respect within my own family, while the family of my husband treated me with respect. I had lots of fun and didn't want to go back to my parent's house at all.* (p: 5)

For one of the participants, marriage was a way to achieve her life goals in a peaceful life. She stated,

*When I was single, it was so bad. My parents were very strict with me; I couldn't even choose my field of study. My wish was to study and become a dentist. That was why I married in order to reach my dreams in a peaceful life.* (p: 7)

The participants believed that they could achieve their personal independence through marriage and acquiring the spouse identity. One of them said,

*I thought marriage could be a new life for me and I could establish my own family too. The marriage is starting an independent life. I have the opportunity to make my own decisions. I manage my own time; I personally have control over all my plans and wishes in my life.* (p: 12)

#### 3.3.2. Emotional Needs

Satisfaction of emotional needs was another reason to persuade the participants to early marriage. They believed that marriage could give them the feeling of being loved. One of the participants stated,

*For the first time in my life, I felt I was going to be in love. I was experiencing emotional feelings toward the opposite sex. This experience was a new good and welcome thing for me. I really needed to be loved by someone.* (p: 14)

#### 3.3.3. Sexual Needs

Based on the participants' beliefs, marriage was a way to satisfy their sexual needs. In this regard, two participants insisted that “*I agreed to get married at that age, because it protected me from falling into depravity. Every girl at this age has sexual needs which could be satisfied through marriage”* (p: 10);* “I am a beautiful girl and many boys were attracted to me. I taught [sic] if I didn't get married, it is possible to fall into the trap [sex outside of marriage]”* (p: 7).

According to some participants, satellite was one of the factors that reinforced their sexual desires. One of the participants mentioned,

*Neither at home nor at school anyone had not taught us anything about sex. I had to learn these things from the satellite…  Some of the things that I saw [in the satellite] increased my sexual drive.* (p: 11)

## 4. Discussion

The current study aimed to explore the determinants of early marriage from married girls' perspectives in Iranian setting. The study results revealed that the participants perceived marriage as an unexpected and stressful event because they were unprepared to accept the roles and responsibilities of an early marriage. Similarly, many studies have shown that marriage forces teenage girls to accept new responsibilities for which they are often not ready physically and psychologically [22–24]. In this study, although most participants intended to postpone their marriage until completion of their education, some factors such as cultural family structure, low authority, and response to needs influenced the participants' decision-making and propelled them to marry. Indeed, these factors exerted the hidden forces that persuaded the participants of early marriage. This concept is different from the enforced marriage that is discussed in the literature [6, 8, 10].

According to the results, family structure was one of the basic factors that propelled the girls to early marriage. In the literature, some dimensions of family structure have been recognized including the family as a system, family norms, roles, communication, the balance of power within the family system, and intergenerational aspects [25]. In Iran as an Islamic country, marriage is a valuable event [15] and is strongly recommended on religious, moral, social, and psychological grounds [26]. In this cultural context, the idea that marriage seems as the only way to obtain an identity has remained unchanged among three generations of Iranian women. Regardless of the great diversity in marriage patterns in some regions and the level of education and achievements in other aspects of life in women, marriage itself is fundamental to the social identity of all women, and force on women to marry persists [15]. Consequently, early marriage has remained common and it is still encouraged by Iranian culture [12, 13, 27].

Scholars maintain that cultural and traditional issues as well as socioeconomic factors are the major forces determining age at marriage in Iran [28]. In the present study, for the participants' families, early marriage was a norm transmitted to the participants as the next generation. Moreover, religious beliefs and socioeconomic difficulties led the participants' families to prefer that their daughters should marry soon. In poor families, girls also preferred to marry for escaping from socioeconomic difficulties. Similarly, the results of Matlabi and colleagues' study indicated that marriage for some Iranian girls was considered as a great way to escape from restrictions of their families [6]. Some studies from other countries also revealed that girls from lower socioeconomic status families were more likely to get married earlier than their counterparts from higher socioeconomic status families [29]. Also, according to UNICEF, poverty has been recognized as the key motivation behind the practice of early marriage [30].

Based on the Islamic religious doctrine, when a person gets married, he indeed perfects half of his religion [31]. Therefore, it can be considered that marriage promotes human spiritual maturity. However, it does not mean to enforce people to marry when they are not ready physically or psychologically: “Allah does not impose upon any soul a duty but to the extent of its ability” (Quran, 2:286). In the current study, regardless of religious doctrine, families encouraged their children to marry while the girls were insisting on not being able to make decisions independently due to being too young and lack of sufficient knowledge and skills. Thus, In Iranian culture, it seems that sociocultural values anticipated the religious values to rationalize early marriage. Although, based on religious teachings, marriage is revoked without couple's agreement and consent [32], the families appear to use the sociocultural and economic factors as excuse to convince their children to marry early.

In the current study, the participants believed that they were not mature enough and had no sufficient autonomy in decision-making for their marriage. Therefore, they could not overcome their parents' authority in regard to the benefits of early marriage. It appears to be a form of subtle or hidden forced marriage. Tremayne also believes that obligations to the family have remained untouched and continue to be main determinants in any decision made related to marriage. In such an instance, the word coercion seems inappropriate because it is not perceived as such but rather as accomplishing a full obligation to the family. Furthermore, the only view for a girl from a traditional family a few decades ago was to get married [15].

Low autonomy due to lack of adequate life skills including decision-making, problem-solving, negotiation, and critical thinking skills played the major role in propelling the girls to early marriage. Life skills are a group of psychosocial competencies and abilities for adaptive and positive behavior that enable individuals to deal effectively with the challenges of everyday life [33]. The results revealed that although all participants were educating until marriage, they had not learned essential life skills and were not empowered enough to make proper decisions for their marriage. Due to lack of sufficient life skills, most of the participants were not able to anticipate the consequences of early marriage and also had not sufficient autonomy in marriage-related decision-making. Therefore, they had to allow their parents to decide for their marriage. Evidence has shown that the high level of literacy in Iran has not led automatically to the empowerment of girls and education without life skills has not added any apparent real value to their lives [15]. In Iranian society, even though early marriage has been a common practice [13], families and educational institutions appear to have not put sufficient effort in empowering girls to prepare for marriage and accept marital responsibilities. Furthermore, young people have insufficient access to information on these matters, whether from parents, teachers, or health services [4, 15, 34].

One of the other factors that propelled the participants to accept early marriage was response to their social, emotional, and sexual needs. The participants viewed marriage as a way to achieve the independence and social identity as an essential part of their transition to adulthood. They thought that marriage would bring for them more respect, peace, love, and autonomy in decision-making in their future life. However, they did not have essential substructures to reach their goals and suffered from inadequate life skills. Previous studies have argued that transition to marriage for teenage girls is often associated with limited access to knowledge and impeded autonomy [35], and married girls have very limited ability to make decision about their own health [36]. In addition to meet social and emotional needs, the participants believed that marriage allows them to satisfy their sexual needs. Within Iranian culture, given that extramarital sexual relations are legally and morally prohibited [12], families intend to encourage their children to marry as soon as possible in order to meet their sexual needs and protect their chastity [37]. According to Tremayne, Iran is a country in transition from traditional to modern society and customs related to marriage are no exception. Tremayne portrayed the interface between tradition and modernity and their ensuing paradoxes in Iran [15]. In this transition, the mass media plays an important role in people's beliefs, attitudes, and family values [38, 39]. In the current study, our results revealed that, based on traditional values, some participants and their families had positive attitudes toward early marriage. Other participants had liberal ideas toward the early onset of sexual relationships due to media impacts and they intended to postpone marriage until achieving their educational and vocational goals. Consequently, mass media might have acted as a double edged sword for our participants. Consistent with our results, Kempadoo and Dunn believe that media educates teenage girls about interpersonal relationships and sexual health and influences their social behavior and thinking. It can also stimulate them via erotic visual images, music, and pornographic movies [40].

## 5. Conclusion

The study results revealed that the participants intended to postpone their marriage but some determinants such as family structure, low autonomy in decision-making, and response to social, emotional, and sexual needs propelled them to early marriage.

Our data highlight underlying factors at various levels associated with marriage-related decision-making process among teenage girls. These findings have implications for policymakers, planners, and health practitioners to develop culturally sensitive programs and interventions tailored to the needs of teenage girls. These programs should emphasize girls' empowerment for making proper decisions and preparing them for marriage at the appropriate manner and time. Given that early marriage is a multifactorial problem, a multidimensional and intersectoral approach should be considered to develop and implement effective and comprehensive programs aimed at raising awareness among families and communities regarding the negative consequences of early marriage. In addition, in order to understand issues related to early marriage, much more qualitative research is needed to address young people perceptions in different cultural contexts.

## Figures and Tables

**Table 1:** Demographic characteristics of participants.

Age of teen pregnant (mean year/month)	16.1
Age of husband (mean year/month)	22.6
Differences between ages of participants and their husband (mean year/month)	6.11
Educational level of participants	
Illiterate	0
Elementary school	0
Junior high school	11
Senior high school	4
Educational level of husband	
Illiterate	0
Elementary school	0
Junior high school	3
Senior high school	3
Diploma	5
Academic	4
Occupation status of participants	
Employed	0
Household	15
Living with father-/mother-in-law	
Yes	13
No	2
Length of marriage (mean/month)	10
Duration of interviews (mean)	87 (min.)

**Table 2:** Categories and subcategories.

Categories	Subcategories
Family structure	Socioeconomic difficulties
	Cultural family values
	Religious beliefs

Low autonomy in decision-making	Inappropriate decision-making skills
	Inadequate problem-solving skills
	Insufficient negotiation skills
	Lack of critical thinking skills

Response to needs	Social needs
	Emotional needs
	Sexual needs
